# Why geese benefit from the transition from natural vegetation to agriculture

**DOI:** 10.1007/s13280-016-0879-1

**Published:** 2017-02-18

**Authors:** Anthony D. Fox, Kenneth F. Abraham

**Affiliations:** 1 Department of Bioscience, Aarhus University, Kalø, Grenåvej 14, 8410 Rønde, Denmark; 2 Wildlife Research and Development Section, Ontario Ministry of Natural Resources and Forestry, Peterborough, ON Canada

**Keywords:** Agricultural conflict, Crop damage, Energetic intake-rate, Feeding profitability, Human–wildlife conflict

## Abstract

The energy and nutrient content of most agricultural crops are as good as or superior to natural foods for wild geese and they tend to be available in agricultural landscapes in far greater abundance. Artificial grasslands (fertilised native swards and intensively managed reseeds) offer far superior quality forage and higher intake rates than seminatural or natural grasslands. The availability of such abundant artificial food explains the abandonment of traditional habitats for farmland by geese over the last 50–100 years and favours no reduction in current levels of exploitation of agriculture. Continental scale spatial and temporal shifts among geese undergoing spring fattening confirm their flexibility to respond rapidly to broadscale changes in agriculture. These dramatic changes support the hypothesis that use of agricultural landscapes has contributed to elevated reproductive success and that European and North American farmland currently provides unrestricted winter carrying capacity for goose populations formerly limited by wetlands habitats prior to the agrarian revolution of the last century.

## Introduction

Over the last 50 years, major biodiversity loss in Europe has been associated with farmland habitats, which cover about 45% of its land area (Kleijn et al. 2011). The loss of bird populations associated with farmland habitats has been particularly dramatic, as witnessed in the >50% drop in the EU farmland bird indicator index since 1980 (Gregory et al. 2005; EBCC 2015). The decline in farmland bird populations has been mainly attributed to agricultural intensification that has been supported by the European Common Agricultural Policy, CAP (Donald et al. 2001, 2006; Szép et al. 2012). These declines continue to the present despite policy aimed to restore agricultural biodiversity (Pe’er et al. 2014) and are also prevalent in North America (Reif 2013). In stark contrast to the declining population trajectories of most North American and European avian species associated with farmland, northern hemisphere geese, which now largely winter exclusively on agricultural landscapes in these two continents, generally show increases in abundance (Fox and Madsen 2017). Why have this group of herbivorous birds been so successful at exploiting the food provided by our agriculture when the abundance and diversity of much of the rest of the avian bird community associated with farmland are in such decline? This paper reviews how geese in particular (but other herbivorous waterbirds such as ducks and swans as well, although we do not deal explicitly with these species here) have benefited from the transition from natural foods provided largely by wetland communities to forage on artificial food resources provided by our own food industry. In particular, the review concentrates on finding support for evidence that food quality, in particular energetic and nutritional content, is far higher on farmland than in wetlands and that this, together with the provision of single-species stands and the generation of abundant accessible waste following harvest, provides more profitable foraging, which has attracted geese onto farmed ecosystems. The review seeks to find support for individual benefits from these shifts in terms of reduced time spent feeding and increased rates of fat accumulation and for population benefits in terms of shifts in distribution and contributions to elevated fitness. Finally, we seek evidence that increases in goose abundance on farmland have exceeded associated changes on continuously occupied natural habitats within populations, suggesting that the increase in overall goose abundance is driven by their colonisation of, and expansion into, agricultural habitats.

### Historical changes in land use and agrarian practice

In mediaeval Europe, farmers were already cultivating crops (including cereals and legumes) while domesticated animals (which grazed grasslands and other ecosystems by day) were confined at night specifically to deposit manure on areas in preparation for the next cropping cycle (Slicher van Bath 1963; Loomis 1978). Despite grain yields per unit area less than 15% of those of today (Connor 2013), such landscapes attracted the attentions of foraging wild geese to the degree that the miraculous ability to remove wild geese from crops was rewarded by sainthood (Kear 2001). Although goose depredation of human agriculture is therefore long established, it is evident that the small scale of cultivation, the far higher human population density associated with agriculture of the time and the pressure from hunting on geese would have precluded any major demographic benefit to goose populations of the time in exploiting mediaeval farmland. European agriculture went through a series of troughs in the late mediaeval, seventeenth and nineteenth centuries (Abel 2006), but it was mainly in the twentieth century that large areas of land were physically claimed from waterbodies, wetlands and the sea. The invention of the Haber–Bosch process in 1908 enabled humans to fix atmospheric nitrogen (Galloway et al. 2008), and inorganic fertiliser application suddenly enabled a vast improvement in plant performance and crop yields, and the cultivation of grass and arable crops on even the most peaty and sandy, infertile soils that were formerly heath, bog and moorland (and ironically the subject of current restoration ecology projects, e.g. Kooijmana et al. 2016). Application of nitrogen fertiliser increased sixfold in the Netherlands between 1939 and 1992 (van Eerden et al. 1996) and 4.2-fold between 1960 and 1980 in the USA (USDA ERA 2016), but with relatively little increase since. Although environmental legislation in the European Union has reduced its regional use since then, global nitrogen application per hectare of land has continued to increase, enhancing grass and crop yields (World Bank 2016). From the 1950s until the 1990s, the use of fertilisers, in combination with better tillage techniques, rapid mechanisation, applications of pesticides and the breeding of better cultivars all combined to dramatically increase production in the European arable sector, including more than a doubling in yield of important crops such as wheat, barley and potatoes (van Eerden et al. 1996). Reseeding of grass cultivar leys in combination with fertiliser application implemented since the 1970s has also advanced the production of grasslands, by extending the growing season in spring and autumn, as well as enhancing the quality and quantity of the biomass produced. As a result, heavily fertilised modern cultivars of the commonly cultivated Italian Rye Grass *Lolium perenne* can be sequentially defoliated and still produce high-quality leaf tissue to grazers, including geese, for prolonged periods of the year (Davies 1988; Lestienne et al. 2006).

### Food quality on agricultural and natural habitats

As a result of these multiple improvements in quality and quantity of agricultural crops and forage grasses, the farm landscape now offers a wide range of foods that far exceed natural food resources in wetlands and other habitats formerly exploited by geese as traditional staging and overwintering habitats. The seeds of maize and common cereals such as wheat, barley and oats all contain very high protein, fat and energy content, but low fibre and ash, and as a result are of generally similar or superior quality compared to natural seeds, tubers and stolons of native plants (Table 1). Since waste maize (e.g. Baldassarre et al. 1983), rice (Stafford et al. 2010) and cereal grains (e.g. Madsen 2001; Jensen et al. 2016) are often present in stubble fields in great abundance, their availability post-harvest and even until spring (Sherfy et al. 2011; Madsen et al. 2014) means that this source of artificial food is far more profitable at different stages of the annual cycle compared to natural food. For instance, although the tubers of *Carex* and *Allium* may be profitable in terms of energy content, their comparative rarity and buried nature means that they need to be gleaned from wetland substrates. They also require enhanced handling times to remove excessive substrate and dead (and therefore less nutritious) tissue prior to consumption (Gates et al. 2001) compared to spilled grain gleaned from stubbles. Similarly, cultivated forage (e.g. clover and alfalfa) and grass crops tend to be of similar or superior quality to native species (Table 1). However, single species cultivation offers dense swards of uniform forage that substantially inflates intake rates over mixed swards where efficient foraging requires selection between species on the basis of quality to maximise intake rates (see below).

**Table 1:** Percentage composition (%) and apparent metabolizable energy (AME measured in kcal/g) of foods consumed by Canada geese *Branta canadensis* in southern Illinois and east-central Wisconsin, USA, 1984–1987 (reproduced from Gates et al. 2001, to which the reader is referred for full methods)

Item	% Crude fat	% Crude protein	% Crude fibre	% Ash	AME (kJ g^**−1**^ **)**
Maize	3.6	10.6	2.4	1.7	16.44
Milo	3.1	11.4	2.5	1.8	16.57
Soybean	18.7	41.7	5.8	5.6	12.68
Small grain	2.0	14.7	2.9	2.0	16.11
Grass seed	3.3	12.4	18.3	9.9	10.17
Forb seed	9.4	18.6	20.2	5.4	9.21
*Carex* tuber	6.8	7.9	7.5	6.8	12.59
*Allium* tuber	1.0	12.5	6.0	5.0	10.54
Clover aerial	5.0	19.4	23.3	10.2	10.46
Clover stolon	2.2	3.9	28.2	3.0	11.09
Alfalfa	2.9	21.1	24.2	10.2	10.13
Wheat aboveground	4.4	27.4	17.4	13.3	10.63
Grass aboveground					
Oct–Feb	3.2	10.5	29.9	9.2	9.92
Oct–Dec	2.8	10.1	31.6	9.5	9.71
Mar–Apr	3.6	17.4	25.2	9.4	10.38
Grass root	2.2	3.9	28.2	3.0	11.09
Misc. forb	3.5	31.6	13.0	10.2	11.55
*Equisetum*	2.4	5.3	23.5	18.5	9.29
*Eleocharis*	1.9	10.9	31.5	7.1	10.08

For example, the mid-continent North American population of the lesser snow geese *Chen caerulescens caerulescens* formerly wintered on salt and brackish marshes on the Gulf of Mexico, but initially switched to use irrigated rice fields in Texas and Louisiana and latterly increasingly to other crops (especially maize) further inland and far to the north of their former winter quarters (Glazener 1946; Lynch et al. 1947; Bateman et al. 1988; Abraham et al. 2005). Alisauskas et al. (1988) studied these wintering geese feeding on coastal marsh, rice paddies and agricultural lands (maize) in Louisiana and showed that geese would have to eat 2.4× more dry weight mass of food from a marsh diet derived from natural foods and 4.3× more of the rice (which included grain but also much plant waste) compared to the maize diet on agricultural land to provide existence energy. Maintenance requirements for nitrogen were satisfied (in terms of crude protein intake) in all habitats if geese consumed enough of any of the diets to meet maintenance energy needs. Tinkler et al. (2009) showed that the relative energy content of foods available to light-bellied brent geese *Branta bernicla hrota* feeding at Strangford Lough, Northern Ireland varied from 14 kJ g^−1^ on intertidal *Zostera*, their tradition natural habitats, to 16 kJ g^−1^ on saltmarsh and 21 kJ g^−1^on managed pasture.

Most geese are grazing herbivores and are adapted to exploiting short, high-quality grass swards with relatively low fibre and high digestibility (Fox et al. 2017). Hence, the revolution in grass sward management that has been manifested in the last 50 years has also brought enormous benefits to geese. Grass biomass and quality (especially protein content, although this generally correlates with high-energy digestibility) are easily enhanced by inorganic fertiliser applications which are now a permanent feature of modern agriculture throughout North America and Europe. A wide range of studies show that geese of many species respond positively to such grassland management by increased goose densities and grazing pressure (e.g. Riddington et al. 1997; Vickery and Gill 1999; Hassall et al. 2001; Durant et al. 2004; Bos et al. 2005; Fox et al. 2017). Cereal crops in the early stages of growth exhibit high protein content that often equal or exceed those of pasture grasses, and in the case of autumn-sown crops such as wheat, therefore, now offer feeding geese monoculture stands of high-quality forage throughout the winter in milder temperate climates (van Eerden et al. 1996; Therkildsen and Madsen 1999, 2000). Clearly, new reseeds, as well as growing winter cereals, with regimented ranks of mono-cultured monocotyledonous swards offer far higher intake rates over mixed swards of grass species and forbs of differing quality that require either high level of visual selection or compromise to achieve optimal food intake rates well below that of the best quality species.

### Intake rates

**Fig. 1:**
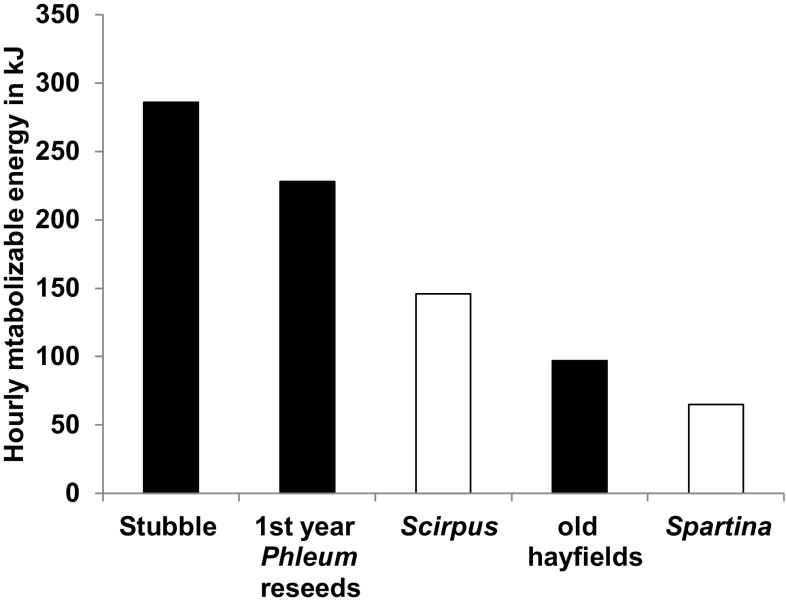
Estimates of hourly metabolisable energy (HME) of spring staging greater snow geese *Chen caerulescens atlantica* feeding on five contrasting southern Quebec habitats, three on agricultural land (stubble, newly reseeded *Phleum pratense* hayfields, and older hayfields, filled histogram columns) and two traditionally used intertidal habitats (*Scirpus americanus* and *Spartina alterniflora* marshes, open histogram columns; from Béchet et al. 2004)

If agricultural products provide better sources of food than natural ecosystems, it is important to show that individuals from the same goose population experience different food, energy and/or nutrient intake rates when feeding on the two sources of food. For instance, pink-footed geese *Anser brachyrhynchus* feeding on new sown barley grains gained almost 16 times more energy (219 J) per peck than those feeding on extensive pastures (14 J, Madsen 1985). However, digestibility of foods differs by up to 100% in studied situations (Hassall and Lane 2005), so it can be inappropriate to use instantaneous intake rates of nutrients as a measure of profitability. Where such correction has been done, it is evident that farmland habitats do offer more rapid accumulation of daily needs. Hence, the time needed for dark-bellied brent geese *Branta bernicla bernicla* to fulfil nitrogen needs was far less on winter wheat (3.7 h day^−1^) compared to semi-natural pasture (mean 5.02 h day^−1^ ± 0.25 SE, range 4.5–5.8) and especially saltmarsh (considered the most natural habitat, mean 11.25 h day^−1^ ± 1.06 SE, range 7.0–14.8, Hassall and Lane 2005). In the case of spring staging greater snow geese *Chen caerulescens atlantica* in southern Quebec, Canada, studies have shown that geese derive 1.5–2 times the metabolisable energy from feeding on new reseeded hayfield swards and 3.5–4.5 times from feeding on spilled grain in stubbles than they can attain from foraging on their traditional intertidal marsh feeding on wild *Scirpus americanus* and *Spartina alterniflora* where grubbing, extraction and handling time add to the challenges of maintaining high food intake rates (Fig. 1; Bédard and Gauthier 1989; Béchet et al. 2004).

### Time spent feeding and the costs of accessing rich food resources

To benefit geese in terms of energetic gain, exploitation of agricultural sources of food must enable the birds to balance their energetic budget in shorter feeding time than on natural foods in a way that provides more time to avoid predation and invest in activities other than foraging. Madsen (1985) showed that the daily net energy intake of pink-footed geese was 2824 kJ day^−1^ on newly sown barley fields where they fed for just 54% of the day compared to 1267 kJ day^−1^ feeding on pastures where they spent 80% of the daylight hours feeding. Although daily energy expenditure (1088 kJ) was lower on pasture than on new sown fields (1280 kJ) because of more frequent disturbance flights, there was far greater net energy gain on barley fields than pasture. This raises another important issue, because clearly attraction to artificial foraging opportunities is only justified energetically if the benefits of accessing such food supplies outweigh the costs (as for instance trading off the benefit of consuming high-energy food against the energetic costs arising from disturbance experienced when accessing that resource, Bédard and Gauthier 1989; Bos and Stahl 2003; Ladin et al. 2011; Clausen et al. 2013). This is especially the case when foraging geese impose a financial burden on agriculture by reducing yields and profitability (Fox et al. 2017), where prolonged intentional disturbance by scaring enhances energy expenditure and leads to higher consumption of grass in situations where reduction of grass consumption is the management objective (Nolet et al. 2016).

### Rate of fat accumulation

During critical periods prior to episodes of energy expenditure, geese must not just balance their energy and nutrient budgets, but must also to acquire body stores, for example, in the form of fat to fuel spring or autumn migration, to invest in reproduction (e.g. in laying or incubating a clutch) or in maintaining condition through wing moult. In such cases, the extra time spent feeding that accrues from feeding on a richer food resource may become crucial in acquiring such stores and may affect the selection between natural and farmland habitats (as in the case of Bewick’s swans *Cygnus columbianus bewickii*, Nolet et al. 2002). The ultimate measure of the effects of individual foraging on different habitat types is to relate the use of agricultural versus natural habitats to the rate of fat and protein stores accumulation of individual geese. Prop and Black (1998) studied spring staging barnacle geese *Branta leucopsis* foraging on intensively farmed, less intensively managed and abandoned islands of the west coast of Norway. On these islands, the main spring food items consumed in hayfields by geese were the native grasses *Festuca rubra* and *Poa* spp., while geese on agricultural fields mainly took reseeded *Phleum pratense*. Ingestion and digestion rates were highest in the agricultural habitat, intermediate on managed islands and lowest on abandoned islands. The accumulation of protein reserves, calculated from nitrogen retention, was highest on managed islands (125 g), intermediate on abandoned islands (104 g) and lowest on agricultural fields (18 g), but accumulation of fat was far higher on agricultural fields (407 g), than on managed (155 g) or abandoned islands (147 g). Despite the difference in calculated protein accumulation, the calculated fat accumulation correlated well with non-destructive field assessments of fat store accumulation (abdominal profile scores), which correlated well with individual use of the habitats on the different islands, but also showed a strong correlation between the probability of raising offspring through to autumn and the fat scores attained by the end of the previous spring staging period (Prop and Black 1998). In the similar case of spring staging Greenland white-fronted geese *Anser albifrons flavirostris* in Iceland, geese feeding exclusively on reseeded non-native *Phleum* hayfields accumulated 654.1 g (mean ± 124.5 SE) of fat during the staging period, which was more than twice that when compared to geese feeding for the same period exclusively on native hayfield grass swards of *Poa* (293.9 g fat ± 68.2 SE) or *Deschampsia* (269.7 ± 77.4 SE) where the more mixed swards also required higher levels of selection to maintain highest intake rates (Nyegaard 1999).

### Shifts in distribution

Geese are inevitably highly mobile when foraging, showing rapid and sensitive shifts in their feeding distribution in response to persistent disturbance, but primarily to availability of the food supply. In the case of agricultural landscapes, this can mean very abrupt movements in response to the harvest, which may suddenly open up a new source of food in the form of spilled grain or maize and equally dramatic loss when these are ploughed in. However, shifts in distribution can also result from major shifts in patterns of cultivation, which also offer a sign of the reliance of particular goose flyway populations to changing patterns of agricultural cultivation. Approximately 45% of North American Atlantic Flyway Canada Geese used to winter in Florida and the Carolinas in the 1950s, but this had fallen to 4% in the late 1980s because of short stopping of up to 70% of the wintering population in Maryland, Delaware and Virginia as a result of the increasing availability of maize there (Malecki et al. 1988). Wild geese are also beginning to show remarkable abilities to learn to exploit new farmland feeding opportunities. For instance, although numbers of pink-footed geese wintering in Britain have increased throughout the range, the most dramatic increases have occurred in the south, associated with feeding on sugar beet *Beta vulgaris* remains post-harvest (Gill et al. 1997). Analysis found no support for buffer effects, degradation of sites elsewhere or any recent increase in local food availability, suggesting cultural learning of the benefits of feeding on a novel agricultural food supply had fuelled the increase (Gill et al. 1997). Changes in agriculture can also affect both the timing and distribution of staging geese, especially in situations where geese can respond to new sources of food. Pink-footed geese have also begun to stay much later in autumn and early winter in west Jutland, Denmark as a result of the increase in maize cultivation there, where they range farther inland from night-time coastal roosts to forage on this highly energy-rich food post-harvest than was also formerly the case (Madsen et al. 2014).

It is evident that geese store nutrients at traditional staging areas during spring for later use during migration and reproduction (e.g. Prop and Black 1998). Staging greater white-fronted geese *Anser albifrons frontalis* at spring staging sites in Nebraska, USA (February–April) and in southern Saskatchewan, Canada (April–May) were studied in 1979–1980 and 1998–1999 by Pearse et al. (2011). In the earlier period, geese accumulated 8.8–17.7 g of lipids per day in spring in Nebraska (Krapu et al. 1995), but during 1998–1999, geese showed no accumulation of fat or protein stores in Nebraska. However, further north on spring migration in Saskatchewan in 1998–1999, they accumulated 11.4 g of fat and 1.6 g of protein per day (SE  =  0.6) over the three-week period (Pearse et al. 2011). In just 20 years, the geese had shifted the locus of energy and nutrient store acquisition from Nebraska to more northern staging sites. This coincided with a dramatic decline in waste maize availability in Nebraska (the primary food source there) and an increase in high-energy pulse crops in Saskatchewan. While this confirms the flexibility of these geese to respond to continental-scale patterns of landscape-level variations in food availability along their flyways caused by changes in agricultural trends and practices, it also confirms their potential vulnerability to such change.

### Contributions to fitness and relative increases on farmland versus natural habitats

Geese may benefit from the effects of shifts from feeding on natural to artificial farmed biotopes in terms of improved nutrition, but does this affect fitness? Ultimate support for our hypothesis that the shift to farmland has driven increases in goose abundance requires the demonstration of elevated demographic benefits accruing to individuals making such a change. Unfortunately, such data are almost impossible to come by, but some indirect support is at least available. Prop and Black (1998) showed positive relationships between use of intensively farmed spring staging areas and fat store accumulation in barnacle geese, as well as between fat stores in spring of marked, followed individuals and their probability of reproductive success assessed in the subsequent autumn. This evidence strongly suggests a direct demographic link between individuals using farmland in spring and their elevated reproductive success compared to geese using other habitats. Fox et al. (2005) showed that Greenland white-fronted geese have shown major habitat shifts since the 1950s from winter use of plant storage organs in natural peatlands to feeding on intensively managed farmland. Declines in local density on, and abandonment of, unmodified traditional wintering habitat and increased reproductive success among those birds wintering on farmland suggest that density-dependent processes were not the cause of the shift in this winter site-faithful population. There was a positive correlation between the mean production of young within each wintering flock and the degree of use of farmland versus natural wetland habitats. Although not constituting proof, this suggests that breeding success was highest among those individuals using improved agricultural habitats compared with those using semi-natural and bog habitats in winter. Because many more geese winter on farmland (where numbers increased rapidly in the 1980s and 1990s) than on wetlands (where numbers remained stable or declined over the same period), farmland feeding flocks contributed many more young than did those exploiting less managed habitats in the population as a whole and were consistently those flocks that showed greatest increase in number (Fox et al. 2005). Béchet et al. (2004) also found that while numbers of greater snow geese were stable or slightly declining on the natural *Scirpus* and *Spartina* habitats, the abundance of those using farmland habitats further away from traditional intertidal areas was increasing with population growth.

Because of the relative complexity of food finding and processing, exploiting natural habitats successfully may demand specialist foraging skills of geese, which potentially results in reduced foraging efficiency among juvenile birds compared to adults in species such as dark-bellied brent geese and Bewick’s swans. This may explain why many large Anatidae maintain parent–offspring relationships through the first winter of life and why families shift from feeding in natural habitats to agricultural fields earlier than adults without offspring. Although parents would probably maintain higher intake rates on natural habitats, the juveniles probably perform better in agricultural habitats (e.g. Inger et al. 2010; Nolet et al. 2014). This may also suggest that in the face of increasing exploitation of farmland habitats, juvenile survival is higher now than in former times when families were constrained to feed on natural habitat types, although direct evidence for this is lacking in the literature.

## Discussion

In this review, we have used several literature examples to show that rapid developments in agriculture in the last 100 years have resulted in a wholesale transformation in the agricultural landscape of the northern temperature regions. We assert that these changes have contributed directly to supporting increasing wintering concentrations of northern breeding geese as well as autumn and spring staging birds migrating between breeding and non-breeding quarters. In particular, these changes have created large areas of land devoted to the production of maize, cereals and legumes which in spilled form after harvest offer geese a rich source of energy and protein compared to wild natural foods which are frequently less abundant, less concentrated and more costly to consume. Modern agriculture also creates extensive monocultures of selectively bred reseeded swards of highly productive grass species and winter-green cereals which are highly palatable to geese. As a result of these farming patterns, compared to those of wetland foraging birds, intake rates are far higher for geese that specialise on agricultural foods because of higher energy and nutrient content, lower associated fibre and reduced handling times. Furthermore, these artificially available foods occur in far greater abundance in the farmland environment which predominates in modern landscapes compared to poorer quality, less abundant natural foods in remnant fragments of traditional (i.e. pre-agricultural) natural habitats. In this respect, it is hardly surprising that geese utilising farmland for foraging are increasing in numbers, while numbers of the same species using natural habitats are stable or declining. Nor is it surprising that it becomes increasingly difficult to scare geese used to feeding on farmland back onto natural refuge habitats, given the discrepancies in potential intake rates between these sources of forage.

However, it must be remembered that there is often considerable seasonal variation in the relative quality and availability of both the natural and agricultural foods selected by geese at any given stage in their annual cycle. This variation can further be affected by relatively rapid food depletion caused by geese as well as plant mortality (e.g. Vickery et al. 1995; Madsen 2001). These patterns make farmland food items more or less profitable in terms of assimilation rates, ingestion rates, nutrient (carbohydrates and/or protein) and digestibility than natural food resources, and these differences may change at different points in the annual cycle (e.g. Ydenberg and Prins 1981; Hassall and Lane 2005; Tinkler et al. 2009).

There have been claims that geese exploiting agricultural food are using the equivalent of human ‘junk food’ and that the refined and selectively bred strains of plants that supply our own food chain cannot be as nutritionally balanced as those previously exploited in more natural times and in less disturbed habitats. It is of course difficult to refute such claims. Prop and Black’s (1998) study of barnacle geese feeding on the most intensively farmed islands in their Norwegian spring staging area showed that they failed to accumulate protein stores to the same degree that their associates did feeding on less intensively managed and abandoned islands. In that case, feeding on agricultural grassland may have yielded higher rates of fat deposition, but did so at the cost of reduced protein accretion due to an unbalanced diet. However, in that study, controlling for year, pairs using different habitats did not differ in their subsequent reproductive success (Prop and Black 1998). There was also no significant difference in the individual reproductive success of spring staging dark-bellied brent geese using either inland agricultural pasture or natural saltmarsh on the Dutch island of Texel (Spaans and Postma 2001). Furthermore, comparisons of amino acid composition of forage, habitat use and dynamics and composition of body stores deposited by barnacle geese feeding on agricultural pasture and in natural salt marsh during spring migratory preparation showed that the content and composition of amino acids were similar among forage from both habitats and appeared equally suitable for protein accretion (Eichhorn et al. 2012). The same study found no relationship between body compositions of geese and their preferred food habitat or any impaired protein accretion among geese feeding on agricultural grassland compared to natural salt marsh.

Although not rich in examples, we see North American goose populations showing very large shifts in spring staging and pre-breeding fattening in response to large-scale changes in farming practice and some European populations starting to do the same (e.g. Madsen et al. 2014). This suggests major flexibility at the population level to adapt to such changes. In addition, in the cases of the barnacle and Greenland white-fronted geese, there does seem to be some evidence of a demographic benefit in terms of enhanced reproductive output associated with individuals that use agricultural foods compared to those continuing to use natural foods. In the case of the Greenland white-fronted goose at least, it would seem that this has been achieved by enhanced reproductive output as well as through major extension of the carrying capacity of their non-breeding habitat, which is no longer likely to be limiting given the exponential increase in the numbers among the populations associated with farmland habitat. Although we lack empirical evidence to support the hypothesis, it also seems likely that the provision of such unlimited sources of artificial food has also directly enhanced survival. This extension of winter habitat to agricultural lands has provided novel sources of food and has enormously increased the potential carrying capacity of winter habitats, which likely imposed some form of density dependence when limited by natural habitats and their lower carrying capacity. Taking all these strands of evidence together, we argue that there is good support for the hypothesis that the exploitation of agricultural croplands and grassland that have come to dominate the non-breeding feeding habitats of most European and North American goose populations has played an important role in their recent increase in abundance.

At the same time, it is important to remember that for some populations, their abundance on agricultural land in recent decades may represent a highly transitory phenomenon. The agricultural industry exists to maximise yields, so increasing effort goes into reducing waste after harvest. For instance, in North America, maize residues left by ever more efficient combine harvesters have dramatically reduced this source of food for geese in the last two decades (Krapu et al. 2004). As a result, mid-continent lesser snow geese are leaner in spring and autumn than 20 years ago (Pearse et al. 2011) and this is affecting their reproductive success (although high survival rates have limited any impact at the population level, G. Krapu unpublished results). Major expansion in soybean cultivation (which has little nutritional value to geese and other waterfowl, Reinecke et al. 1989) in the Great Plains region of North America has reduced the area and availability of other higher energy foods there (Krapu et al. 2004). Climate change is lengthening growing seasons and political intervention (such as the US Energy Independence and Security Act of 2007 which required 10% ethanol in petrol produced from maize) as well as world markets drive agricultural cropping patterns in unpredictable ways that are not always likely to be beneficial for foraging geese.

It would therefore appear that contemporary agriculture has allowed geese to move from the limited carrying capacity of their former natural non-breeding habitats into what appears to be unlimited potential non-breeding habitats (at least for the time being), which has helped support their massive expansion, albeit also aided by climate change on the breeding grounds (e.g. Boyd et al. 1982; Jensen et al. 2008). Because the nutritional and energetic advantages of exploiting these artificial habitats are so great compared to former natural food resources, the genie is unlikely to be easy to return to the bottle in the sense that it will be difficult to encourage non-breeding geese to return to natural habitats while agricultural food resources continue to exist in parallel. Nevertheless, we need to recognise that the ephemeral agricultural landscapes of the present day and the energy and nutrient subsidies that they currently provide to support enormous numbers of arctic breeding geese outside the nesting season may not continue without change in the future. The efficiency and globalisation of agricultural production, the development of new and innovative crops and the effects of market demands and climate change will all interact to radically change the face of farming which will affect the availability of food for geese and ultimately their abundance and distribution. This further underlines the need to strategically manage goose populations around the northern hemisphere to comprehensively take account of the diverse effects of global change, land use, economic, biodiversity and hunting interests as they happen, to safeguard their future well-being.

## References

[CR1] Abel W (2006). Agricultural fluctuations in Europe, from the thirteenth to the twentieth century.

[CR2] Abraham KF, Jefferies RL, Alisauskas RT (2005). The dynamics of landscape change and snow geese in mid-continent North America. Global Change Biology.

[CR3] Alisauskas RT, Ankney CD, Klaas EF (1988). Winter diets and nutrition of midcontinental lesser snow geese. Journal of Wildlife Management.

[CR4] Baldassarre GA, Whyte RJ, Quinlan EE, Bolen EG (1983). Dynamics and quality of waste corn available to postbreeding waterfowl in Texas. Wildlife Society Bulletin.

[CR5] Bateman HA, Joanen T, Stutzenbaker CD, Weller MW (1988). History and status of midcontinental snow geese on their Gulf Coast winter range. Waterfowl in Winter.

[CR6] Béchet A, Giroux J-F, Gauthier G (2004). The effects of disturbance on behaviour, habitat use and energy of spring staging snow geese. Journal of Applied Ecology.

[CR7] Bédard J, Gauthier G (1989). Comparative energy budgets of greater snow geese *Chen caerulescens atlantica* staging in two habitats in spring. Ardea.

[CR8] Bos D, Stahl J (2003). Creating new foraging opportunities for Dark-bellied Brent *Branta bernicla* and Barnacle Geese *B. leucopsis* in spring—insights from a large-scale experiment. Ardea.

[CR9] Bos D, Drent RH, Rubinigg M, Stahl J (2005). The relative importance of food biomass and quality for patch and habitat choice in Brent Geese *Branta bernicla*. Ardea.

[CR10] Boyd H, Smith GEJ, Cooch FG (1982). The lesser snow geese of the eastern Canadian Arctic: their status during 1964–79 and their management from 1981 to 1990. Canadian Wildlife Service Occasional Paper.

[CR11] Clausen KK, Clausen P, Fox AD, Fælled CC, Madsen J (2013). Varying energetic costs of Brent Geese along a continuum from aquatic to agricultural habitats: The significance of habitat-specific energy expenditure. Journal of Ornithology.

[CR12] Connor DJ (2013). Organically grown crops do not a cropping system make and nor can organic agriculture nearly feed the world. Field Crops Research.

[CR13] Davies A, Jones MB, Lazenby A (1988). The regrowth of grass swards. The grass crop: The physiological basis of production.

[CR14] Donald PF, Green RE, Heath MF (2001). Agricultural intensification and the collapse of Europe’s farmland bird populations. Proceedings of the Royal Society of London. Series B.

[CR15] Donald PF, Sanderson FJ, Burfield IJ, van Bommel FPJ (2006). Further evidence of continent-wide impacts of agricultural intensification on European farmland birds, 1990–2000. Agriculture, Ecosystems & Environment.

[CR16] Durant D, Fritz H, Duncan P (2004). Feeding patch selection by herbivorous *Anatidae*: The influence of body size, and of plant quantity and quality. Journal of Avian Biology.

[CR17] EBCC indicators (2015). www.ebcc.info/indicators2015.html.

[CR18] Eichhorn G, Meijer HAJ, Oosterbeek K, Klaassen M (2012). Does agricultural food provide a good alternative to a natural diet for body store deposition in geese?. Ecosphere.

[CR19] Fox AD, Madsen J (2017). Threatened species to super-abundance: The unexpected international implications of successful goose conservation. Ambio.

[CR20] Fox AD, Elmberg J, Tombre I, Hessel R (2017). Agriculture and herbivorous waterfowl: A review of the scientific basis for improved management. Biological Reviews.

[CR70] Fox AD, Madsen J, Boyd H, Kuijken E, Norriss DW, Tombre IM, Stroud DA (2005). Effects of agricultural change on abundance, fitness components and distribution of two arctic-nesting goose populations. Global Change Biology.

[CR21] Galloway JN, Townsend AR, Erisman JW, Bekunda M, Cai Z, Freney JR, Martinelli LA, Seitzinger SP (2008). Transformation of the nitrogen cycle: Recent trends, questions and potential solutions. Science.

[CR22] Gates RJ, Caithamer DF, Moritz WE, Tacha TC (2001). Bioenergetics and nutrition of Mississippi Valley Population Canada Geese during winter and migration. Wildlife Monographs.

[CR23] Gill JA, Watkinson AR, Sutherland WJ (1997). Causes of the redistribution of pink-footed geese *Anser brachyrhynchus* in Britain. Ibis.

[CR24] Glazener WC (1946). Food habits of wild geese on the Gulf Coast of Texas. Journal of Wildlife Management.

[CR25] Gregory RD, van Strien A, Voříšek P, Gmelig Meyling AW, Noble DG, Foppen RPB, Gibbons DW (2005). Developing indicators for European birds. Philosophical Transactions of the Royal Society of London Series B.

[CR26] Hassall M, Lane SL (2005). Partial feeding preferences and the profitability of winter-feeding sites for brent geese. Basic and Applied Ecology.

[CR27] Hassall M, Riddington R, Helden A (2001). Foraging behaviour of Brent Geese, *Branta b. bernicla*, on grasslands: Effects of sward length and nitrogen content. Oecologia.

[CR28] Inger R, Harrison XA, Ruxton GD, Newton J, Colhoun K, Gudmundsson GA, McElwaine G, Pickford M (2010). Carry-over effects reveal reproductive costs in a long-distance migrant. Journal of Animal Ecology.

[CR29] Jensen RA, Madsen J, O’Connell M, Wisz MS, Tømmervik H, Mehlum F (2008). Prediction of the distribution of arctic-nesting pink-footed geese under a warmer climate scenario. Global Change Biology.

[CR30] Jensen GH, Tombre IM, Madsen J (2016). Environmental factors affecting numbers of pink-footed geese *Anser brachyrhynchus* utilising an autumn stopover site. Wildlife Biology.

[CR31] Kleijn D, Rundlöf M, Scheper J, Smith HG, Tscharntke T (2011). Does conservation on farmland contribute to halting the biodiversity decline?. Trends in Ecology & Evolution.

[CR32] Kear J (2001). Three medieval accounts of agricultural damage by wild geese. Archives of Natural History.

[CR33] Kooijmana AM, Cusella C, van Mourika J, Reijmana T (2016). Restoration of former agricultural fields on acid sandy soils: Conversion to heathland, rangeland or forest?. Ecological Engineering.

[CR34] Krapu GL, Brandt DA, Cox RR (2004). Less waste corn, more land in soybeans, and the switch to genetically modified crops: Trends with important implications for wildlife management. Wildlife Society Bulletin.

[CR35] Krapu GL, Reinecke KJ, Jorde DG, Simpson SG (1995). Spring staging ecology of mid-continent greater white-fronted geese. Journal of Wildlife Management.

[CR36] Ladin ZS, Castelli PM, McWilliams SR, Williams CK (2011). Time energy budgets and food use of Atlantic brant across their wintering range. Journal of Wildlife Management.

[CR37] Lestienne F, Thornton B, Gastal F (2006). Impact of defoliation intensity and frequency on N uptake and mobilization in *Lolium perenne*. Journal of Experimental Botany.

[CR38] Loomis RH (1978). Ecological dimensions of medieval agrarian systems: an ecologist responds. Agricultural History.

[CR39] Lynch JJ, O’Neil T, Lay DW (1947). Management significance of damage by geese and muskrats to Gulf Coast marshes. Journal of Wildlife Management.

[CR40] Madsen J (1985). Relations between change in spring habitat selection and daily energetics of pink-footed geese *Anser brachyrhynchus*. Ornis Scandinavica.

[CR41] Madsen J (2001). Can geese adjust their clocks? Effects of diurnal regulation of goose shooting. Wildlife Biology.

[CR42] Madsen J, Bjerrum M, Tombre IM (2014). Regional management of farmland feeding geese using an ecological prioritization tool. Ambio.

[CR43] Malecki RA, Sheaffer SE, Enck JW (1988). Influence of agricultural land use changes on wintering Canada Geese in the Atlantic Flyway. Transactions of the North-eastern Section of the Wildlife Society.

[CR44] Nolet BA, Bevan RM, Klaassen M, Langevoord O, Van Der Heijden YGJT (2002). Habitat switching by Bewick’s swans: Maximization of average long-term energy gain?. Journal of Animal Ecology.

[CR45] Nolet BA, Gyimesi A, van Lith B (2014). Lower foraging efficiency of offspring constrains use of optimal habitat in birds with extended parental care. Ibis.

[CR46] Nolet BA, Kölzsch A, Elderenbosch M, van Noordwijk AJ (2016). Scaring waterfowl as a management tool: How much more do geese forage after disturbance?. Journal of Applied Ecology.

[CR47] Nyegaard, T. 1999. Fedtakkumulering og energibudget hos forårsrastende Grønlandske blisgæs i Island. Unpublished M.Sc thesis, University of Copenhagen, Denmark. (In Danish with English summary).

[CR48] Pearse AT, Alisauskas RT, Krapu GL, Cox RR (2011). Changes in nutrient dynamics of midcontinent greater white-fronted geese during spring migration. Journal of Wildlife Management.

[CR49] Pe’er G, Dicks LV, Visconti P, Arlettaz R, Báldi A, Benton TG, Collins S, Dieterich M (2014). EU agricultural reform fails on biodiversity. Science.

[CR50] Prop J, Black JM (1998). Food intake, body reserves and reproductive success of Barnacle Geese *Branta leucopsis* staging in different habitats. Norsk Polarinstitutt. Skrifter.

[CR51] Reif J (2013). Long-term trends in bird populations: A review of patterns and potential drivers in North America and Europe. Acta Ornithologica.

[CR52] Reinecke KJ, Kaminski RM, Moorhead DJ, Hodges JD, Nassar JR, Smith LM, Pederson RL, Kaminski RM (1989). Mississippi Alluvial Valley. Habitat management for migrating and wintering waterfowl in North America.

[CR53] Riddington R, Hassall M, Lane SJ (1997). The selection of grass swards by Brent Geese *Branta b. bernicla*: Interactions between food quality and quantity. Biological Conservation.

[CR54] Sherfy MH, Anteau MJ, Bishop AA (2011). Agricultural practices and residual corn during spring crane and waterfowl migration in Nebraska. Journal of Wildlife Management.

[CR55] Slicher van Bath BH (1963). The Agrarian history of Western Europe: A.D. 500–1500.

[CR56] Spaans B, Postma P (2001). Inland pastures are an appropriate alternative for salt-marshes as a feeding area for spring-fattening Dark-bellied Brent Geese *Branta bernicla*. Ardea.

[CR57] Stafford JD, Kaminski RM, Reinecke KJ (2010). Avian foods, foraging and habitat conservation in world rice fields. Waterbirds.

[CR58] Szép T, Nagy K, Nagy Z, Halmos G (2012). Population trends of common breeding and wintering birds in Hungary, decline of long- distance migrant and farmland birds during 1999–2012. Ornis Hungarica.

[CR59] Therkildsen OR, Madsen J (1999). Goose grazing selectivity along a depletion gradient. Ecography.

[CR60] Therkildsen OR, Madsen J (2000). Energetics of feeding on winter wheat versus pasture grasses: a window of opportunity for winter range expansion in the Pink-Footed Goose *Anser brachyrhynchus*. Wildlife Biology.

[CR61] Tinkler E, Montgomery WI, Elwood RW (2009). Foraging ecology, fluctuating food availability and energetics of wintering brent geese. Journal of Zoology.

[CR62] Van Eerden MR, Zijlstra M, van Roomen M, Timmerman A (1996). The response of Anatidae to changes in agricultural practice: Long term shifts in the carrying capacity of wintering waterfowl. Gibier Faune Sauvage.

[CR63] Vickery JA, Gill JA (1999). Managing grassland for wild geese in Britain: A review. Biological Conservation.

[CR64] Vickery JA, Sutherland WJ, Watkinson AR, Rowcliffe JM, Lane SJ (1995). Habitat switching by dark-bellied brent geese *Branta b. bernicla* (L.) in relation to food depletion. Oecologia.

[CR65] USDA ERA. 2016. Fertilizer use and price. Washington DC: United States Department of Agriculture Economic Research Service. Accessible at: http://www.ers.usda.gov/data-products/fertilizer-use-and-price.aspx.

[CR66] World Bank. 2016. Fertilizer consumption (kilograms per hectare of arable land).Washington DC: World Bank Group. Accessible at: http://data.worldbank.org/indicator/AG.CON.FERT.ZS.

[CR67] Ydenberg RC, Prins HHT (1981). Spring grazing and the manipulation of food quality by Barnacle Geese. Journal of Applied Ecology.

